# Evaluation of a tele-refraction model to address the lack of human resources in Kenya

**DOI:** 10.1371/journal.pone.0352780

**Published:** 2026-07-30

**Authors:** Shadrack Muma, Jyoti Naidoo, Khathutshelo Percy Mashige, Tshubelela Sello Simon Magakwe

**Affiliations:** 1 Kaimosi Friends University, School of Health Sciences, Kaimosi, Kenya; 2 African Vision Research Institute, University of KwaZulu-Natal, College of Health Sciences, Durban, South Africa; Alexandria University Faculty of Medicine, EGYPT

## Abstract

**Background:**

Uncorrected Refractive Errors (URE) constitute a significant public health concern in Kenya, where limited availability of qualified eye care professionals continues to impede equitable access to refractive services. This shortage is particularly acute in rural and underserved regions, resulting in significant unmet visual health needs. The implementation of telemedicine; specifically, a tele-refraction model utilizing low-cost, portable devices, has emerged as a viable and cost-effective approach to decentralize service delivery, enhance accessibility, and mitigate the burden of URE at the primary healthcare level.

**Methods:**

This non-inferiority diagnostic accuracy study was conducted in Machakos.

County, Kenya, to evaluate the performance of the OneSight EssilorLuxottica Foundation’s ClickCheck™ tele-refraction model in comparison with conventional face-to-face retinoscopy, which served as the reference (gold) standard. A total of 92 patients (184 eyes) were examined. The tele-refraction procedure entailed a trained technician performing objective refraction using the ClickCheck™ device, followed by subjective refinement under the real-time supervision of a remote optometrist via telecommunication. Statistical analyses assessed the agreement between the final subjective prescriptions obtained from both methods, focusing on the clinical conformity rate for spherical equivalent (SE), cylindrical power, and power vector components within a strict clinical tolerance limit of ±0.50 diopters (D).

**Results:**

The study demonstrated a high level of clinical concordance between the telerefraction model and the conventional gold-standard refraction method. The mean difference in spherical equivalent (SE) for objective refraction was minimal (+0.08 D), indicating negligible bias between the two techniques. Importantly, the final subjective prescriptions exhibited excellent clinical interchangeability, with 93.01% of cases falling within the ± 0.50D tolerance for SE and 94.41% within the same tolerance for cylindrical power magnitude. Furthermore, for clinically significant astigmatism, axis alignment showed complete conformity within the 10 degrees tolerance range across all cases.

**Conclusion:**

The tele-refraction model using the ClickCheck™ device, combined with remotely guided subjective refraction, demonstrated accuracy, reliability, and non-inferiority when compared with conventional in-person refraction. These findings substantiate the clinical validity of tele-refraction, which holds the potential to mitigate the acute shortage of eye care professionals and to enhance equitable access to refractive error correction services among underserved populations in Kenya.

## Background

It is estimated that more than six million individuals in Africa live with uncorrected refractive errors, predominantly in remote, socioeconomically disadvantaged, and rural communities [1]. In Kenya, where approximately 71% of the 47 million inhabitants reside in rural areas, access to refractive error services remains critically limited. Only an estimated 10–20% of those requiring vision correction can obtain and afford such services [2]. Although no comprehensive population-based studies on refractive errors have been published in Kenya, existing estimates suggests that approximately 6.39% of the population is affected by uncorrected refractive errors (3). Moreover, prevalence rates reported from underserved rural districts range from 5.2% to over 17.2% [3].

Access to eye care services in Kenya is constrained by several barriers, including a shortage of trained eye health professionals, high service costs, inadequate diagnostic and treatment equipment, and unequal geographical distribution of eye care facilities, particularly disadvantaging populations in remote and rural regions where transportation remains a major challenge [4]. Additionally, socioeconomic factors such as the prioritization of basic living expenses over healthcare, disability-related challenges, lack of awareness about eye conditions and treatment outcomes, and entrenched cultural beliefs, fears, and mistrust of medical interventions further exacerbate disparities in access to timely and effective eye care services [5].

A major challenge confronting Kenya’s eye care sector is the acute shortage of qualified optometrists. Kenya has approximately 400 trained optometrists; however, only about 56 are currently registered with the Optometrists Association of Kenya, and the profession remains unregulated by the government [2]. Consequently, access to optometric services is severely limited, particularly in rural and underserved regions. The demand for optometric services continues to grow, driven by an aging population, an increasing prevalence of eye diseases, and greater public awareness of the value of routine eye examinations [1]. Optometrists in Kenya are employed across diverse settings, including private practices, government health facilities, non-governmental clinics, and specialized eye units, where they provide comprehensive visual assessments, diagnose and manage ocular conditions, and prescribe corrective lenses and low-vision devices [6]. However, the lack of statutory regulation exposes patients to potential risks, as individuals seeking care from optical outlets may be attended to by unqualified personnel or receive incomplete ocular health evaluations.

The limited number of trained eye care professionals, coupled with the concentration of ophthalmic services within referral hospitals, contributes to overcrowding and prolong waiting times. This situation further discourages timely health-seeking behaviour, particularly among socioeconomically disadvantaged individuals who often prioritize occupational responsibilities over healthcare needs [1]. In response to these systemic challenges, the Kenyan government, in collaboration with several partner organizations, has initiated optometry training programmes across different counties to strengthen the national eye health workforce [6]. However, a major barrier persists in the form of limited access to modern diagnostic equipment and technology, with many practising optometrists continuing to rely on outdated instruments, thereby constraining the quality and efficiency of service delivery.

Despite these challenges, the optometry profession in Kenya holds significant potential to improve population health outcomes. Through targeted investments in human resource development, the adoption of innovative technologies, and the strengthening of healthcare infrastructure, optometry can broaden service coverage and substantially enhance the accessibility and quality of eye care among underserved communities [7].

The rising prevalence of uncorrected refractive errors underscores the urgent public health challenge requiring the implementation of cost-effective and sustainable interventions. Despite growing demand for refractive services, the limited availability of trained eye care personnel remains a major constraint, particularly in rural and underserved regions. Recent research by Muma S et al (2024) reports that Kenya currently has approximately 400 optometrists, 151 ophthalmologists, 40 ophthalmic clinical officers, and 176 ophthalmic clinical officers/cataract surgeons serving an estimated population of 53 million people [8]. In comparison, the World Health Organization (WHO) recommends a minimum ratio of one refractionist per 50,000 individuals [9], underscoring a significant human-resource gap in

Kenya’s eye health workforce. This shortage necessitates the development of innovative, scalable, and cost-efficient strategies to improve access to refractive services, particularly within rural and resource-limited settings [8].

### The proposed solutions to providing availability and access to refractive error services

In recent years, diverse strategies have been adopted to enhance access to refractive error services within underserved populations. The accelerated advancement of telecommunication and digital technologies has significantly influenced the delivery of healthcare services. Telemedicine, defined as the remote provision of healthcare through information and communication technologies, has emerged as a transformative approach to overcoming geographical and infrastructural barriers between patients and healthcare providers. Examples exist where this innovation has played a pivotal role in expanding access to essential medical care, particularly in remote and resource-limited settings [10].

For instance, in Ethiopia, the implementation of tele-ophthalmology has been advocated as a feasible strategy to address the critical shortage of ophthalmologists, estimated at approximately one ophthalmologist per 1,200,000 individuals [10]. In Kenya, the application of mobile health (mHealth) technologies has demonstrated considerable promise in extending eye care services to underserved and remote communities. A notable example is Peek Vision, a smartphone-based visual acuity assessment tool, which has been shown to reduce preventable blindness by enabling early detection of refractive errors and improving compliance with referral pathways [11].

Additionally, existing reports indicate that approximately 89% of rural South Africa, Mauritius, Kenya, and Malawi have internet connectivity, indicating that the implementation of telemedicine in these regions may encounter minimal infrastructural implementation barriers [12]. The utilisation of telehealth technologies has demonstrated potential to enhance operational efficiency, reduce healthcare expenditure, and improve patient outcomes, even within resource-limited settings [13]. Nevertheless, despite increasing global interest in telerefraction as a viable strategy to mitigate the burden of uncorrected refractive errors, the literature continues to exhibit substantial gaps concerning its reliability, accuracy, feasibility, and long-term sustainability [14–16]. Although several studies have examined the effectiveness of tele-optometry, empirical evidence comparing tele-refraction with conventional in-person refraction in diverse, real-world environments remains limited, particularly within low-resource contexts [14]. Uncertainties persist regarding its diagnostic accuracy in detecting subtle refractive errors, operational feasibility in settings with inadequate digital infrastructure, and overall acceptability among practitioners and patients [17,18]. Furthermore, critical aspects related to sustainability such as cost-effectiveness, integration into existing health systems, and the long-term maintenance of tele-refraction services; warrant further investigation [18]. Despite the growing potential of tele-refraction to expand access to refractive care, the indispensable leadership role of optometrists must be underscored, as their clinical expertise is essential for accurate interpretation of findings, quality assurance, and management of complex visual conditions beyond basic refractive correction.

A recent advancement in telemedicine, tele-refraction, has emerged as an innovative and effective approach for improving access eye care services in remote and underserved regions. This technology facilitates the accurate assessment of refractive errors, visual acuity, and other key optometric parameters without requiring patients to travel to conventional eye care centres. In the tele-refraction model, a trained technician conducts the preliminary refraction procedures using digital or automated systems, after which a remote optometrist reviews the results in real time, confirming the diagnosis and issuing the final prescription [19–21].

### The OneSight EssilorLuxottica Foundation ClickCheck™ device approach

This research aims to assess the accuracy of refractive error measurements obtained by a trained technician using the ClickCheck™ device, developed by the OneSight EssilorLuxottica Foundation, in conjunction with real-time supervision by a remote optometrist. The OneSight EssilorLuxottica tele-refraction model employs an on-demand digital platform that facilitates interaction among the primary vision care provider, the trained technician, the patient, and a qualified optometrist. During the procedure, the optometrist remotely monitors the refraction process via live video consultation, verifies and refines the measurements recorded by the technician, and provides final authorization of the prescription through the platform [14].

In this model, trained technicians initially obtained the patient’s case history and unaided visual acuity, followed by assessment of baseline refractive status using the ClickCheck™ device [14].

### ClickCheck™ device and measurement procedure

ClickCheck™ is a portable, self-contained instrument designed to estimate refractive error through manual adjustment of a dial on a plastic tube. Baseline refractive status was assessed using the ClickCheck™ device, a handheld, portable, battery-free instrument designed for rapid estimation of spherical refractive error in community and primary care settings. The device consists of a cylindrical plastic housing approximately 15 cm in length, incorporating an eyepiece, an internal optical lens system, an adjustable diopter control ring, and an enclosed fixation target. It is supplied with a protective storage case to facilitate transport and field use.

**Fig 1:**
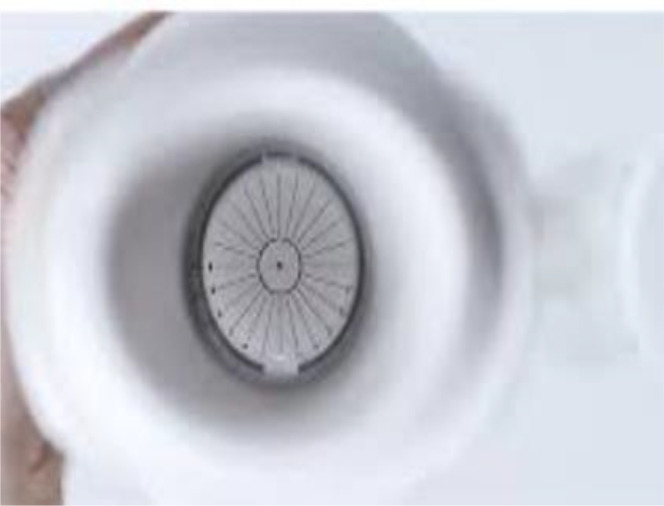
Internal fixation target as seen through the eyepiece of ClickCheck™, demonstrating the high-contrast radial pattern used for clarity judgment [22].

Internally, the device contains a high-contrast radial fixation target positioned within a Badal lens system (Fig 1). This optical configuration allows controlled manipulation of vergence while maintaining constant retinal image size, thereby minimizing magnification cues and enabling reliable subjective determination of refractive clarity.

### Optical principle

ClickCheck™ operates on the Badal optometer principle, whereby movement of an internal lens assembly alters the vergence of light entering the eye without changing angular retinal image size. Rotation of the external diopter ring translates the lens system along the optical axis, modifying accommodative demand while preserving image scale. This permits estimation of spherical refractive error based on the patient’s perception of maximal image clarity.

#### Measurement procedure.

**Fig 2:**
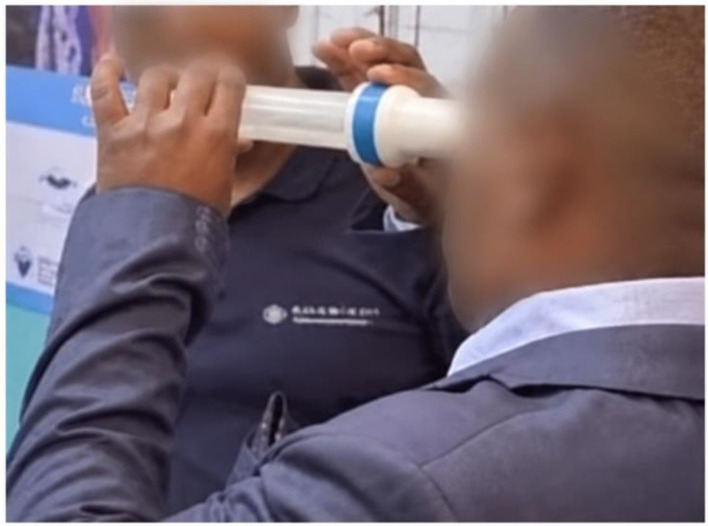
Proper monocular use of ClickCheck™, showing alignment with the visual axis and occlusion of the fellow eye during measurement.

The device is used monocularly, with the fellow eye occluded (Fig 2). Participant should sit comfortably and be instructed to hold the device at eye level, aligned with the visual axis. The patient is asked to look through the eyepiece at the internal fixation target and slowly rotate the diopter control ring until the target appeared maximally clear. If blur reoccurred, participant reverse the dial slightly to refine the endpoint. The diopter value corresponding to the position of greatest sustained clarity is recorded as the estimated spherical refractive error. The procedure is repeated for the contralateral eye.

#### Clinical considerations.

Given that measurements are performed without cycloplegia; accommodation may have influence on estimates, particularly in younger participants. ClickCheck™ provides an estimate of spherical refractive error and does not independently measure cylindrical power or axis. The device is intended for screening and triage rather than full diagnostic refraction.

#### Accuracy and validation.

The clinical validity of ClickCheck™ has been demonstrated by Joseph et al. [22], who reported good agreement between ClickCheck™ estimates and standard subjective refraction, with mean differences within clinically acceptable limits, supporting its utility for refractive screening and task-shifting models in low-resource settings. However, this validation was conducted outside Africa. Differences in refractive error epidemiology, population characteristics, examiner skill mix, and health system infrastructure may influence device performance and limit the direct generalizability of findings to African settings.

Context-specific validation is therefore warranted to assess the agreement, reliability, and operational feasibility of ClickCheck™ within an African population. Generating locally derived evidence will strengthen external validity, inform policy and implementation decisions, and support appropriate integration of the device into primary eye care and school eye health programs in resource-constrained African contexts.

### Implementation and evaluation of the tele-refraction model

Following the acquisition of baseline refractive measurements, the trained technician establishes a connection with a remote optometrist through the tele-refraction platform. The optometrist remotely accesses the patient’s clinical data, providing real-time guidance to the technician throughout the subjective refraction procedure. During this process, the optometrist visually evaluates the patient and refines the refractive findings to determine the final prescription. The optometrist’s diagnosis is regarded as the gold standard for the patient’s definitive prescription. Patients requiring additional assessment or management are subsequently referred to a secondary eye care facility for comprehensive evaluation [23].

Despite the growing global attention in tele-refraction as an innovative approach to improving access to refractive care, its utilisation remains limited. Only a few studies have directly compared tele-refraction outcomes with those obtained through face-to-face refraction [23]. To date, no published research in Kenya has evaluated the accuracy and real-time clinical effectiveness of tele-refraction conducted under the supervision of a remote optometrist. In response to this knowledge gap, the present project aims to implement and assess the feasibility and diagnostic accuracy of tele-refraction within the Kenyan public sector and to propose evidence-based strategies for its future integration. The project will incorporate structured training on the use of the ClickCheck™ device for objective refraction combined with remotely guided subjective refraction and will compare tele-refraction findings with results obtained by onsite optometrists to determine the validity, reliability, and clinical applicability of this approach.

### EyeConnect telerefraction platform

#### Platform overview.

**Fig 3:**
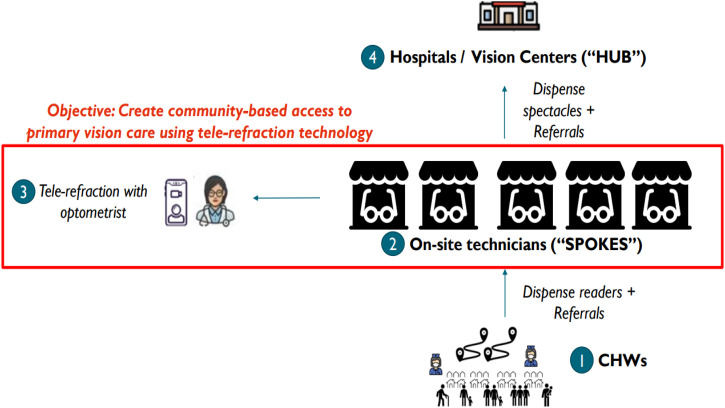
Tele-refraction process.

The EyeConnect Telerefraction application developed by the OneSight EssilorLuxottica Foundation was used to facilitate real-time tele-refraction consultations between trained technicians and remotely located optometrists. The platform was designed to support remote refractive services in underserved and resource-limited settings through telemedicine technology (Fig 3).

#### Operational workflow.

The EyeConnect platform operates through internet-enabled smartphones or tablets and supports live audiovisual communication between the technician and the supervising optometrist. During the consultation process, trained technicians initially obtained patient demographic information, clinical history, visual acuity measurements, and objective refractive findings using the ClickCheck™ device. The refractive findings were subsequently uploaded to the EyeConnect platform for review by a remotely located optometrist. The supervising optometrist remotely guided the technician through subjective refraction procedures in real time, including trial lens selection and refinement of refractive power, while simultaneously monitoring patient responses through the platform. Following completion of the subjective refinement process, the optometrist validated and authorized the final spectacle prescription digitally through the application.

#### Telemedicine features.

The platform supports several telemedicine functions that facilitate remote refractive care delivery. These include real-time audiovisual communication, remote supervision of visual acuity assessment, live guidance during subjective refraction, digital entry and transmission of refractive findings, and remote validation and authorization of prescriptions. The platform also enables secure communication between technicians and optometrists and allows continuity of care through reconnection with the same optometrist in cases where consultations are interrupted by connectivity challenges.

#### Technological requirements and availability.

The EyeConnect platform requires stable internet connectivity and operates on Android and iOS mobile devices, including smartphones and tablets. The application is designed for field-based use and enables tele-refraction consultations in community and primary healthcare settings where access to optometrists may be limited. The EyeConnect Telerefraction application is publicly available on both Android and iOS platforms through the Google Play Store and Apple App Store.

#### Aim of the study.

To evaluate the accuracy and clinical effectiveness of a tele-refraction model using the ClickCheck™ device combined to remotely guided subjective refraction using associated tele-optometry technology, in comparison with conventional in-person refractive examination methods.

#### Definitions of terms.

*Tele-refraction* – A remote method of determining a patient’s refractive error using digital tools and communication technology.

*Refractive error* – A vision problem caused by the eye’s inability to focus light properly on the retina, leading to blurred vision

*ClickCheck™ device* – A portable tool developed by the OneSight EssilorLuxottica Foundation for estimating refractive errors.

*Telemedicine* – The use of digital communication technologies, such as video calls and mobile applications, to provide healthcare services remotely, reducing the need for in-person visits.

*mHealth (Mobile Health)* – A subset of telemedicine that involves using mobile devices like smartphones, tablets, and health applications to support healthcare delivery, including patient monitoring and diagnosis.

*Retinoscope* – A handheld instrument used by optometrists to measure refractive errors by observing how light reflects from the retina when shined into the eye.

*Objective refraction* – A method of measuring refractive errors without requiring subjective input from the patient

*Subjective refraction* – A vision assessment method where the patient provides feedback while looking through different lenses to determine the best prescription for clear vision

*Badal optometer principle* – An optical principle that maintains a constant image size while varying the target distance and stimulus to accommodation

*Vision impairment* – A significant reduction in vision that cannot be corrected fully with standard eyeglasses, contact lenses, or medical treatment, often affecting daily activities

## Methods

The study employed a non-inferiority diagnostic accuracy design and was conducted prospectively study in Yatta Sub-County and Kathiani Sub-County of Machakos County, Kenya. These two sub-counties collectively have an estimated population of 284,473 individuals comprising 49% males and 51% females. Machakos County was purposively selected due to its demographic diversity, encompassing both urban and rural populations, thereby providing a representative setting to evaluate the applicability and performance of tele-refraction services across varied contexts. The rural segments of the country are characterised by limited access to eye care, attributed to a shortage of optometrists, geographical barriers, and extended travel distances to healthcare facilities, highlighting the need for alternative, technology-based service delivery models.

Machakos presents a demographic profile characterised by an ageing population with an increasing demand for refractive error correction amid limited access to specialised eye care services. Previous eye health assessments conducted in the region reported a notably high prevalence of uncorrected refractive errors, underscoring the urgent need for innovative solutions such as tele-refraction. The selection of Machakos County as a study site thus provides a representative and contextually relevant environment for evaluating the feasibility and effectiveness of tele-refraction technology within Kenya’s broader eye health landscape. The study adhered to the ethical principles outlined in the Declaration of Helsinki, with comprehensive information sheets and consent provided to all participants prior to obtaining informed consent for participation.

Participants were informed about the anonymous and confidential handling of their data, which would be used solely for research purposes. They were also advised of their right to voluntarily withdraw from the study at any stage without any adverse consequences. Recruitment was conducted over a ten-day period, from 4 August 2025 to 13 August 2025, during community-based vision screening outreaches conducted in the study area. Study participants comprised community members aged 18 years and above who voluntarily presented for vision screening services during the outreach activities. Participants were recruited consecutively at the screening sites after being informed about the study objectives and procedures. Eligible individuals who met the inclusion criteria and provided informed consent were enrolled into the study.

### Sample size.

The study aimed to compared refractive error measurements obtained through tele-refraction with those obtained by trained optometrists during conventional face-to-face examinations. A discrepancy exceeding ±0.5 diopters (D) between the two methods was considered clinically unacceptable (Huang et al., 2022). Eyes with clinically significant astigmatism were defined as those demonstrating cylindrical power of ≥0.75 diopters (D). This threshold was selected based on previous refractive error literature indicating that astigmatism of this magnitude is generally considered visually and clinically relevant for refractive correction. The sample size was determined to estimate the proportion of cases in which refractive findings from trained technicians corresponds with those of optometrists within the ± 0.5 D threshold. Assuming a precision of ±10% and a 95% confidence interval (CI), the required sample size was calculated to be 184 eyes. The calculation further assumed maximum variance and incorporated a design effect of 2 to account for clustering or correlated observations.

### Data collection.

Trained technicians initially obtained baseline demographic and clinical history from each participant. Subsequently, uncorrected distance visual acuity (UDVA), pinhole visual acuity, and best-corrected distance visual acuity (BCVA) were assessed and documented. Objective refraction, including spherical and cylindrical powers and axis, was measured using the ClickCheck™ device.

The resulting data were entered into the tele-refraction application, which facilitated a secure remote connection with a licensed optometrist. Through this platform, the optometrist reviewed the transmitted data in real time and provided step by step guidance to the technician in completing the subjective refraction procedure.

The optometrist provided the trained technician with instructions regarding the initial selection of trial lenses to be placed on the trial frame for the patient. During the procedure, the optometrist controlled and adjusted the digital visual acuity display while the technician interchanged the trial lenses as directed. As the technician refined the trial lens powers, the optometrist concurrently updated the refraction parameters within the application until optimal visual acuity attained. Upon completion of the refraction process, the optometrist recorded the final prescription in the application, including the BCVA. The prescription was then formally authorised by the optometrist, enabling the technician to dispense the corresponding spectacles. In cases where additional diagnostic evaluation was deemed necessary, the optometrist referred the patient to the nearest ophthalmic hospital for further assessment.

Trained technicians were permitted to engage with optometrists fluent in the local language to facilitate effective communication during consultations. In instance where teleconsultation was disrupted, technicians had the capacity to reconnect with the same optometrist to maintain continuity of care. Each participant subsequently underwent an in-person objective refraction conducted by a different optometrist using a Heine BETA 200 streak retinoscope, which served as the reference (gold standard) measure. To minimise potential bias, the face-to-face optometrist was blinded to the tele-refraction findings. The final refractive prescription was determined following completion of a subjective refraction during in-person examination.

Data obtained from this evaluation were analysed to determine the proportion of cases in which tele-refraction yielded prescriptions consistent with those obtained through conventional refraction methods. This analysis facilitated a systematic validation of tele-refraction as a viable and evidence-based model for delivering refractive services in underserved populations.

### Inclusion and exclusion criteria

#### Patients.

- Participants included individuals aged 18–75 years who presented to the base hospital for ocular examination and met the inclusion criteria of ± 6 diopters spherical and ± 2 diopters cylindrical correction. Only those with clear ocular media and no evidence of corneal, retinal or clinically apparent ocular pathology were enrolled in the study.
- Patients exhibiting a dull fundal reflex, such as that observed in high myopia, vitreous haemorrhage, or cataract were excluded to ensure optical measurements and minimize confounding from ocular media opacities. *Trained technicians*:
- All technicians received standardized training on visual acuity assessment, ClickCheck^TM^ device, trial lens application, operation of the EyeConnect tele-refraction platform and prescription interpretation. All technicians underwent a 2-day standardized training program (16 hours total) delivered by licensed optometrists. The training covered: (1) visual acuity assessment using distance and near charts, including patient positioning, illumination control, monocular testing, and recording standards; (2) theoretical and practical use of the ClickCheck™ device, including optical principles, device handling, endpoint determination, and common sources of error; (3) trial lens application, including lens selection, verification of spherical power, and patient comfort optimization; and (4) prescription interpretation, including understanding spherical equivalent, referral thresholds, and criteria for spectacle dispensing. Training included didactic instruction, supervised hands-on practice, competency assessments, and standardization exercises to ensure inter-technician reliability prior to field deployment; (5) operation of the EyeConnect tele-refraction platform, including patient data entry, secure transmission of examination findings, remote communication with supervising optometrists, troubleshooting connectivity issues, and maintaining data confidentiality
- Prior to inclusion in the study, technicians were required to demonstrate proficiency by completing a minimum of 20 refractions using the tele-refraction platform accurately and efficiently.

#### Optometrist for face-to-face examination.

Two optometrists, each possessing a minimum of two years of clinical experience, conducted online face-to-face examinations as remote practitioners. Prior to the commencement of the study, a pilot assessment involving 20 patients was performed to standardise procedures and ensure consistency in examination standards between the participating optometrists.

### Statistical analysis

Agreement between tele-refraction and face-to-face refraction measurements was assessed using power vector analysis rather than conventional sphere, cylinder, and axis comparisons. Refractive data were transformed into spherical equivalent and Jackson cross-cylinder components J0 and J45 following the method described by Miller [24], enabling vector-based comparison of refractive error independent of axis orientation.

Agreement between modalities was evaluated using Bland–Altman analysis for spherical equivalent values. Mean bias and 95% limits of agreement (LoA) were calculated and graphically displayed. In addition, intervals of clinical relevance (±0.50 D and ±1.00 D) were overlaid on the plots to facilitate clinical interpretation of measurement differences. Similar Bland–Altman analyses were conducted for J0 and J45 components.

This approach is widely accepted in refractive error research and provides a more accurate and clinically meaningful assessment of agreement between refractive measurement methods than correlation-based metrics. Additionally, comparative analyses were conducted to evaluate the mean differences in these parameters between the two main groups (telerefraction and face-to-face refraction), as well as among subgroups stratified according to their conformity levelsPrior to conducting inferential analyses, the assumptions underlying the use of independent sample t-tests were assessed. Normality of refractive error measurements was evaluated using the Shapiro–Wilk test together with visual inspection of histograms and Q–Q plots. Homogeneity of variances was assessed using Levene’s test. As some refractive variables demonstrated non-normal distributions, non-parametric methods were additionally considered. Consequently, where assumptions for parametric testing were not met, the Mann–Whitney U test was used as the appropriate alternative to compare refractive outcomes between groups.

#### Data management.

Research data will be securely retained for a period of five years following completion of the study. Upon expiration of this retention period, all data will be permanently destroyed to prevent any possibility of retrieval r reconstruction. To maintain confidentiality, participants’ identities were anonymised during data collection through the assignment of unique identification codes.

#### Ethical consideration.

This study was performed in accordance with the Declaration of Helsinki, and has been approved by Maseno University Ethics Review Committee (MUERC/0031/25). Informed consent to participate in the study was obtained from all participants.

## Results demographic characteristics

A total of 92 participants (184 eyes) completed the study and were included in the final analysis. Of these, 59 were female (64.1%) and 33 were male (35.9%). The mean age of participants was 36.6 years.

There was no statistically significant association between participants’ age group and gender (p = 0.2200). Both male and female participants were proportionally represented across the various age categories, indicating an absence of gender-related bias in the sample and supporting the representativeness of the study population. The distribution of participants by age group and gender.

### Refractive error counts and percentages

**Fig 4:**
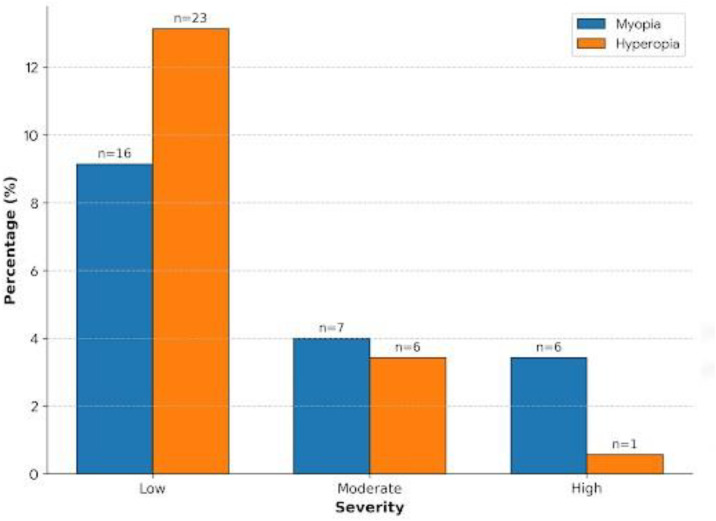
Distribution and severity of refractive errors.

The study analyzed the distribution of refractive errors by categorizing eyes into low, moderate, and high severities based on the spherical equivalent (SE). The classification criteria were defined as low (0.50 to 3.00 D), moderate (3.25 to 6.00 D), and high (> 6.00 D). Myopia and hyperopia were the most prevalent conditions, affecting 16.57% and 17.14% of the eyes, respectively. The detailed distribution of these conditions across severity levels as per the Eyeconnect tele-refraction is presented in Fig 4. This stratification allows for a more rigorous evaluation of the telerefraction model’s performance in both low-diopter cases and more complex, high-power refractive errors.

Female participants demonstrated a mean SE of −0.69 ± 2.78 D, while male participants demonstrated a mean SE of −0.50 ± 2.83 D. As refractive error data were not normally distributed, comparison between groups was performed using the Mann–Whitney U test, which showed no statistically significant difference between biological sex groups (p = 0.761).

### The comparison between the ClickCheck objective refraction and the retinoscopy objective refraction

The mean difference between the two objective methods was minimal (+0.08D), indicating that measurements obtained were closely aligned on average. The 95% limits of agreement for retinoscopy ranged from -0.62D and 0.79D, whereas for the objective ClickCheck method, they ranged from −0.54D to 0.32D. The narrower range observed with ClickCheck reflects a substantially higher degree of agreement and improved clinical interchangeability between the two objective assessment techniques. The detailed comparative results are presented in Table 1.

**Table 1:** Comparison between ClickCheck and retinoscopy.

Analysis Type	Mean Difference (Bias) (D)	95% Limits of Agreement (D)	Pearson r
Objective (ClickCheck vs. Retinoscopy)	+0.02	−0.54 to 0.32	0.809
Subjective (ClickCheck vs. Retinoscopy)	+0.08	−0.62 to 0.79	0.966

A very strong positive correlation was observed between the subjective spherical equivalent (SE) measurements obtained using ClickCheck and those derived from retinoscopy (r = 0.966). This correlation was substantially stronger than that observed for the objective comparison (r = 0.609).

### Accuracy of ClickCheck objective vs. Retinoscopy objective

Agreement between the objective refractive measurements obtained using ClickCheck™ and conventional retinoscopy was assessed using Bland–Altman analysis of spherical equivalent (SE) values. The mean difference (bias) between the two methods was small (+0.02 D), indicating minimal systematic measurement error between ClickCheck™ and retinoscopy. The 95% limits of agreement ranged from −0.54 D to +0.32 D, demonstrating a narrow distribution of measurement differences and good clinical agreement between the two objective refraction techniques.

Most measurements (79.34%) fell within the clinically acceptable ±0.50 D interval, while all remaining measurements were within ±1.00 D. Because refractive error data were not normally distributed, comparison between methods was additionally assessed using the non-parametric Wilcoxon signed-rank test, which demonstrated no statistically significant difference between the two objective refraction methods (p = 0.620).

**Fig 5:**
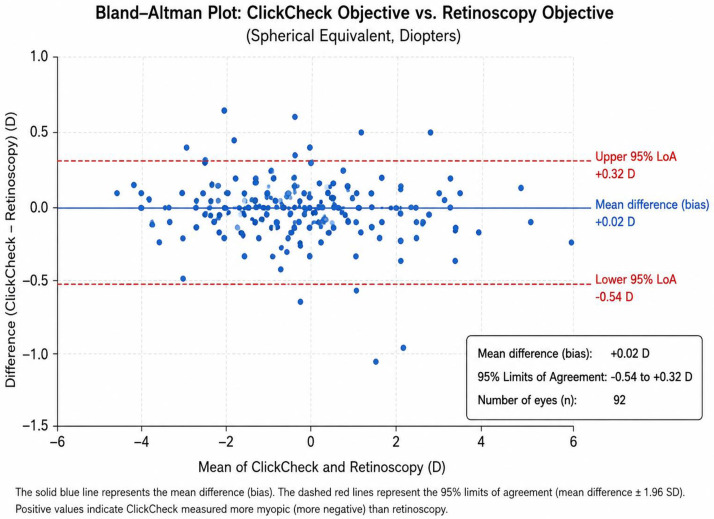
Bland-Altman Plot: ClickCheck vs. Retinoscopy.

The Bland–Altman plot illustrating agreement between ClickCheck™ objective refraction and retinoscopy is presented in Fig 5.

### Conformity rates for cylindrical power

**Fig 6:**
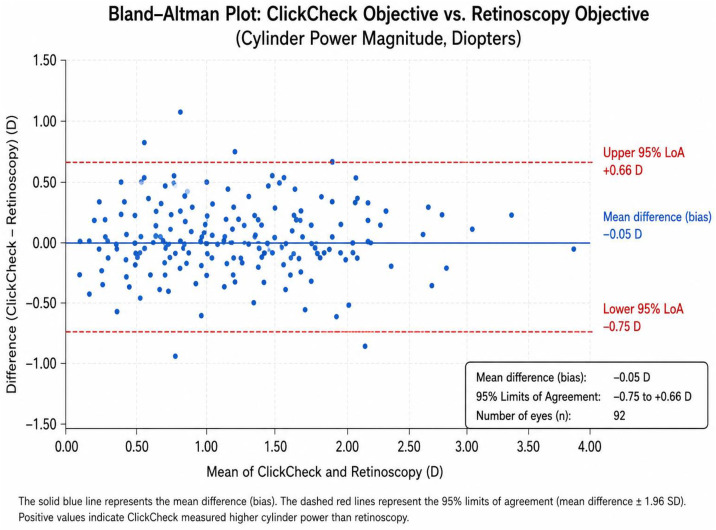
Bland-Altman Plot: ClickCheck Vs. Retinoscopy.

Approximately two thirds (67.39%) of the objective ClickCheck cylinder power measurements corresponded with the objective retinoscopy values within a tolerance of ±0.50D tolerance, while an additional 32.61% fell within the broader ± 1.00D tolerance range, as shown in Fig 6. These findings indicate a high level of agreement between objective measurement techniques, suggesting that both methods are comparably accurate and may be used interchangeably. Furthermore, analysis of the cylinder power magnitude revealed no statistically significant difference between the mean values obtained using objective ClickCheck and objective retinoscopy (p = 0.720). This implies that neither method demonstrates a systematic tendency to overestimate or underestimate cylinder power relative to the other as shown in Supplementary 1 (S1 File).

### Objective refraction: axis conformity rate

For eyes presenting with clinically significant astigmatism, a high level of agreement (87.56%) was observed between the objective ClickCheck and objective retinoscopy methods. Only 12.44% of eyes demonstrated an axis discrepancy exceeding 10 degrees, representing a minimal deviation of limited clinical significance. Statistical analysis using the Mann–Whitney U test demonstrated no statistically significant association between age group and axis conformity (p = 0.1689).

### Subjective Spherical Equivalent (SE), cylinder power and axis conformity

Analysis of the final subjective prescriptions revealed that 93.01% of cases demonstrated agreement within a ± 0.50 D criterion for spherical equivalent (SE). For cylindrical power, 94.41% of cases fell within the ± 0.50 D tolerance. Among the 44 eyes identified with clinically significant astigmatism, 100% of the subjective axis measurements derived from the ClickCheck™ system and retinoscopy agreed within a ± 10-degree tolerance as shown in Supplementary 2 (S2 File).

## Discussion

This study evaluated the clinical performance and reliability of a tele-refraction model utilizing the ClickCheck™ device as an alternative to traditional retinoscopy in a resource-limited setting in Kenya. The primary objective was to determine if this remote-supervised model could provide refractive results non-inferior to the gold standard, thereby addressing the significant gap in human resources for eye health.

The findings demonstrate high clinical concordance between the tele-refraction model and conventional face-to-face retinoscopy. Specifically, 93.01% of the final subjective prescriptions for spherical equivalent fell within the ±0.50 D clinical tolerance. Notably, this accuracy remained robust across a diverse distribution of refractive errors, including low, moderate, and high ametropes. By stratifying the results by severity, the study confirms that the device’s utility extends beyond simple refractive errors, maintaining high agreement even in cases of high myopia and hyperopia.

Furthermore, the objective measurements provided by the ClickCheck™ device demonstrated high agreement with objective retinoscopy, with the majority of measurements falling within a narrower and clinically meaningful tolerance interval of ±0.50 D.. This level of agreement, together with the high concordance observed for astigmatic axis measurements within a 10-degree tolerance, suggests that the tele-refraction model shows potential as a feasible and reasonably reliable approach for refractive assessment, although the clinical implications of larger refractive differences should be interpreted with caution. The findings of this study underscore the critical role of human resources in eye health, as the tele-refraction model implemented herein depended extensively on the expertise of trained technicians and remotely based optometrists to ensure accurate and reliable service delivery. The mean participant age of 36.6 years indicates a predominantly young, economically population, emphasizing the need for accessible and time-efficient refractive services that minimize both travel distance and associated costs.

Findings from this study indicate that the tele-refraction model, utilizing the ClickCheck™ device in combination with a distance-guided subjective refraction, indicates that it could effectively bridge the service gap in rural counties such as Machakos. By providing a clinical concordance rate of 93.01% within ±0.50 D for spherical equivalent, the model demonstrates that accurate refractive services can be decentralised from major urban centers to rural primary healthcare levels. This approach directly addresses the accessibility constraints identified in rural populations, where the lack of resident optometrists traditionally necessitates significant patient travel for basic refractive assessments [22]. The ability to maintain high diagnostic accuracy across various severities of refractive error from low to high ametropia further supports the implementation of this remote-supervised model as a reliable solution for enhancing equitable access to eye care.

Findings from this study indicate that trained optical technicians, when supervised remotely, can effectively mitigate this gap by providing reliable tele-refraction services [6]. The present study demonstrates that a tele-refraction model utilizing portable technology and remote optometric supervision can effectively augment existing eye care services. By achieving a high level of clinical agreement, with 93.01% of subjective prescriptions falling within ±0.50 D of the gold standard, this model shows promise as a practical approach for decentralizing refractive care and expanding access to refractive services in underserved settings.

Notably, the observed level of agreement was maintained across participants with varying degrees of ametropia, including moderate and high refractive errors, suggesting that the tele-refraction process may be applicable across a broad spectrum of refractive severities.. This suggests that while traditional eye care facilities remain essential, the tele-refraction approach may serve as a reliable primary-level intervention, potentially reducing the diagnostic burden on mission-based and public hospitals by enabling management of refractive errors at the community level. This suggests that while traditional eye care facilities remain essential, the tele-refraction approach may serve as a reliable primary-level intervention, potentially reducing the diagnostic burden on mission-based and public hospitals by enabling management of refractive errors at the community level. The present study identified myopia, hyperopia, and astigmatism among participants presenting for vision screening, reflecting the range of refractive conditions encountered within the study sample. These findings support the relevance of decentralized and accessible refraction services, particularly in community-based settings where access to comprehensive eye care may be limited. The results of this study suggest that the integration of tele-refraction models into primary healthcare settings could enhance the sustainability of eye care coverage in Kenya. Unlike intermittent outreach programs, which are often limited by logistical constraints and long-term viability, a permanent or semi-permanent tele-refraction initiative at the community level allows for consistent follow-up and service delivery. Similar technology-enabled vision center models have demonstrated potential in various lowresource settings by reducing the travel burden on patients and providing a more stable framework for refractive care. While the infrastructure and technological requirements differ across regional contexts, the high clinical concordance of 93.01% observed in this study supports the transition toward decentralized, technology-supported refractive services as a means to achieve universal eye health coverage.

The integration of digital technology in eye care, particularly through tele-refraction utilizing portable refractive assessment tools and remote optometric supervision, represents a promising advancement in refractive service delivery. Findings from the present study revealed a high level of agreement between tele-refraction and conventional face-to-face retinoscopy, with minimal differences observed in both spherical equivalent and axis measurements. These outcomes align with the findings of Kapur et al. [14], who reported in a similar study comparing these two protocols that 84.6% of cases demonstrated concordance within ±0.5 D for spherical correction and that 82% achieved comparable best-corrected visual acuity to traditional in-person refraction. The concordance between the two studies substantiates the reliability of tele-refraction performed under remote optometrist supervision and supports the use of ClickCheck™ as an accurate tool for refractive assessment in resource-constrained environments.

The implementation of tele-refraction hubs connected to remotely based optometrists presents a feasible strategy for continuous service provision in resource-limited environments. The observed agreement across participants with varying refractive severities, including moderate and high ametropia, suggests that this approach may support the integration of digital health solutions to enhance refractive service delivery at the primary healthcare level.

### Limitations of the study

Face-to-face retinoscopy conducted by qualified optometrists remains the gold standard for objective refraction. However, inter-practitioner variability in retinoscopy technique may have introduced some measurement variability between examiners. Objective refraction findings were therefore followed by subjective refraction refinement, which reflects standard clinical practice for optimizing refractive prescriptions. Additional study limitations include the facility- and outreach-based sampling approach, which may limit generalizability to the broader population, as well as the relatively short study duration and the absence of long-term follow-up to assess patient adaptation and satisfaction with prescribed corrections..

In conclusion, this study demonstrates that the ClickCheck™ tele-refraction model provides refractive outcomes with high clinical concordance to conventional retinoscopy. These findings support the potential of remote-supervised refraction to expand access to accurate eye care services in resource-limited settings.

## Supporting information

S1 File TR data for the study. (XLS)

S2 File Subjective and objective data. (XLSX)
